# Microsatellite Instability and MMR Genes Abnormalities in Canine Mammary Gland Tumors

**DOI:** 10.3390/diagnostics10020104

**Published:** 2020-02-14

**Authors:** Faiz Muhammad Khand, Da-Wei Yao, Pan Hao, Xin-Qi Wu, Asghar Ali Kamboh, De-Ji Yang

**Affiliations:** College of Veterinary Medicine, Nanjing Agricultural University, Nanjing, Jiangsu 210095, China; drfaizkhand@yahoo.com (F.M.K.); yaodawei@njau.edu.cn (D.-W.Y.); 2017207032@njau.edu.cn (P.H.); 2014107110@njau.edu.cn (X.-Q.W.); drasgharkamboh@yahoo.com (A.A.K.)

**Keywords:** canine mammary gland tumors, microsatellite instability, polymorphism, MMR system

## Abstract

Early diagnosis of mammary gland tumors is a challenging task in animals, especially in unspayed dogs. Hence, this study investigated the role of microsatellite instability (MSI), MMR gene mRNA transcript levels and SNPs of MMR genes in canine mammary gland tumors (CMT). A total of 77 microsatellite (MS) markers in 23 primary CMT were selected from four breeds of dogs. The results revealed that 11 out of 77 MS markers were unstable and showed MSI in all the tumors (at least at one locus), while the other markers were stable. Compared to the other markers, the ABC9TETRA, MEPIA, 9A5, SCNA11 and FJL25 markers showed higher frequencies of instability. All CMT demonstrated MSI, with eight tumors presenting MSI-H. The RT-qPCR results revealed significant upregulation of the mRNA levels of *cMSH3*, *cMLH1*, and *cPMSI*, but downregulation of *cMSH2* compared to the levels in the control group. Moreover, single nucleotide polymorphisms (SNPs) were observed in the *cMSH2* gene in four exons, i.e., 2, 6, 15, and 16. In conclusion, MSI, overexpression of MMR genes and SNPs in the MMR gene are associated with CMT and could be served as diagnostic biomarkers for CMT in the future.

## 1. Introduction

Genomic instability, a hallmark of cancer, is generally characterized by DNA mismatch repair (MMR) defect, that leads to microsatellite instability (MSI). Therefore, cell MSI plays a critical role in the genesis of mammary gland tumors. Maintenance of genomic stability ensures the inheritance of a complete copy of genetic material in the daughter cells. Moreover, during replication, cells may develop multiple forms of mutations in several genes, such as chromosomal rearrangements, as well as a gain or a loss of part(s) of or the entire chromosome [1].

Repetitive sequences of 1–6 nucleotide base pairs in DNA are known as microsatellites. In addition, alterations in microsatellites are an important form of genomic instability, referred to as MSI. These tandem repeat-sequences are dispersed across the genomes of eukaryotes, usually in noncoding regions. Inactivation of the MMR system results in mutations, particularly, highly repetitive sequences. Additionally, the distribution of microsatellites throughout the genome leads to MSI [2,3].

More than 2000 identified canine microsatellite (MS) markers have been identified and considered useful genetic markers for genetic mapping [4]. MSI most likely occurs during the replication of genetic material, and any errors introduced during this process result in the addition or deletion of a base pair. These genomic changes may cause abnormalities in cell division and hence lead to an imbalance between cell growth and death or ultimately cause cancer. In addition, the MMR system maintains the integrity of the genome [5], whereas MMR-deficient cells exhibit a mutator phenotype in microsatellites with a higher incidence of mutations [6].

Moreover, MutS-α (*MSH2* and *MSH3*) and protein complexes of MutL are the basic recognition complexes of the MMR system. The MutS-α complex is involved in the recognition of base–base mismatches and small insertion or deletion loops, whereas the MutS-β complex corrects larger loop mispairs. Furthermore, an efficient MMR system requires the binding of MutL protein complexes to MutS-α or MutS-β [7]. Germline mutations associated with MMR system proteins, either hereditary [8] or sporadic [9,10], lead to tumor development. The *cMSH2* gene plays a central role in mismatch recognition, and some studies suggest that there are polymorphisms in *cMSH2* [11,12] in humans.

Although an ovariohysterectomy can provide protection against mammary tumorigenesis if performed early in life, canine mammary gland tumors (CMT) constitute the most common tumors in intact female dogs. Understanding the specific genetic mechanisms in carcinogenesis would be of beneficial for the prevention, diagnosis and treatment of CMT. DNA damage, MMR and MSI are important mechanisms during tumorigenesis. At present, the data regarding the involvement of MSI and MMR gene in canine mammary gland tumors (CMT) are limited. The objective of this study is to evaluate the MSI, MMR genes expression, and polymorphisms in the *cMSH2* gene in CMT, which will provide new potential biomarkers for the diagnosis and treatment of tumors.

## 2. Materials and Methods

### 2.1. Ethical Statement

Tumor samples were collected with the consent of the owners and according to the recommendations published in the Guide for the Care and Use of Laboratory Animals of Jiangsu province (SYXK2017-0027). The Committee on the Ethics of Animal Experiments of Jiangsu province approved the protocol (NJAU-20171019, 10 October 2017).

### 2.2. Tumor Sample Collection

Twenty-three CMT from 23 female dogs of four different breeds were provided by the Teaching Hospital of Nanjing Agricultural University. The age of the patients ranged from 5 to 15 years (9 years mean age). Tumors were collected from the female dogs by mastectomy, followed by confirmation through histopathological evaluation. The adjacent normal mammary glands were also excised during the surgical procedure. The tumor tissues and adjacent normal mammary glands were divided into two parts, one part of which was fixed in 10% formalin solution for histopathological assessment and the other was frozen at −80 °C for DNA and RNA extraction.

### 2.3. Tumor Histopathological Assessment

The tumor tissues samples as well as the normal tissue samples were removed from each dog and fixed into 10% formalin solution. Then, these samples were embedded in paraffin wax via a routine process. All sections were stained with hematoxylin and eosin (H&E) and histopathologically examined using an optical microscope. Mammary tumors were classified according to the classification proposed by Goldschmidt et al. [13].

### 2.4. DNA Extraction and PCR Amplification

DNA was isolated from the frozen samples (tumor and adjacent normal mammary glands) using commercially available kits (Invitrogen USA) according to the manufacturer’s instructions. The quality of the DNA was assessed on 0.8% (w/v) agarose gel by electrophoresis and was quantified with a spectrophotometer. Isolated DNA was diluted (80 ng/L) and stored at −80 °C to determine MSI.

### 2.5. Microsatellite Instability (MSI)

Seventy-seven microsatellite markers in the canine linkage map were used to screen the 23 samples taken from four different dog breeds for MSI evaluation [14,15,16]. The eleven unstable markers are shown in Table 1. The PCR product of each marker was denatured at 95 °C for 5 min with gel loading dye, and immediately put on ice for 5 min prior to loading. Approximately 3 μL vol of the PCR products were separated on 10% polypropylene polyamide gels (1 mm thick). The gels were subsequently stained with AgNO_3_. The bands of each locus were counted and evaluated. The MSI-H group of tumors was defined as having MSI in ≥30–40% of the loci, whereas MSI-L group exhibited MSI in <30% of the loci [17].

### 2.6. Expression Profiling by RT-qPCR Assay

The total RNA was isolated from tumor and adjacent normal mammary gland tissue (50 mg) with TRIZOL (Invitrogen, Carlsbad, CA, USA) according to the manufacturer’s instructions. Approximately 250 ng/µL of the total RNA template was used to synthesize the first-strand cDNA with Prime Script RT Master Mix Perfect Real Time (Takara Co., Otsu, Japan) according to the manufacturer’s instructions. The abundance of RNA transcripts of different genes was estimated using quantitative real-time PCR (RT-qPCR) for gene-specific primers. The primers for *cMSH2, cMSH3, cMLH1*, *cPMS1*, and *GAPDH* (as a housekeeping gene) were designated by primer premier 5.0 software, the information is available in GenBank database NCBI reference sequence (XM_538482.5, XM_022417321.1, XM_534219.6, XM_536002.6 and NM_001003142.2 respectively) (Table 2). The RT-qPCR mixture (20 µL) contained cDNA (2 µL) and the reverse and forward primers (0.4 µM) in the SYBR Green master mix. ABI 7300 Fast Real-time PCR System (Applied Biosystem, USA) was used for amplification, programmed at 95 °C for 15 s of initial denaturation, and annealed for 40 cycles at 95 °C for 5 s, followed by 60 °C for 31 s of primer extension. The *GAPDH* housekeeping gene of the dog was used as an internal Control. The relative value to gene expression was computed on the basis of 2^−ΔΔCT^ (−ΔΔCT = − [(C_T target gene_ − C_T GAPDH_) tumor − (C_T target gene_ − C_T GAPDH_) normal]).

### 2.7. Detection of Exon Mutations of the cMSH2 Gene

The sixteen pairs of primers were designed from the related sequence information available in the GenBank database (NC_006592.3) using Primer Premier 5.0 software to amplify the 16 exons of the *cMSH2* gene. The amplification conditions were the same as mentioned above, except for the annealing temperature, which varied according to each primer used (Table 3). The targeted amplicons from the PCR product of each primer were retrieved from the agarose gel, purified, and cloned into the pGEM-T easy plasmid vector (Promega, Madison, WI, USA). The cloned exons were sequenced on an ABI PRISM 377 capillary sequencer using vector- and exon-specific primers.

### 2.8. Statistical Analysis

One-way analysis of variance (ANOVA) was performed using Predictive Analytics Software 18.0. In addition, Duncan’s multiple-range test was used, with differences considered significant at *p* < 0.05.

## 3. Results

### 3.1. Histopathological Assessment

**Figure 1:**
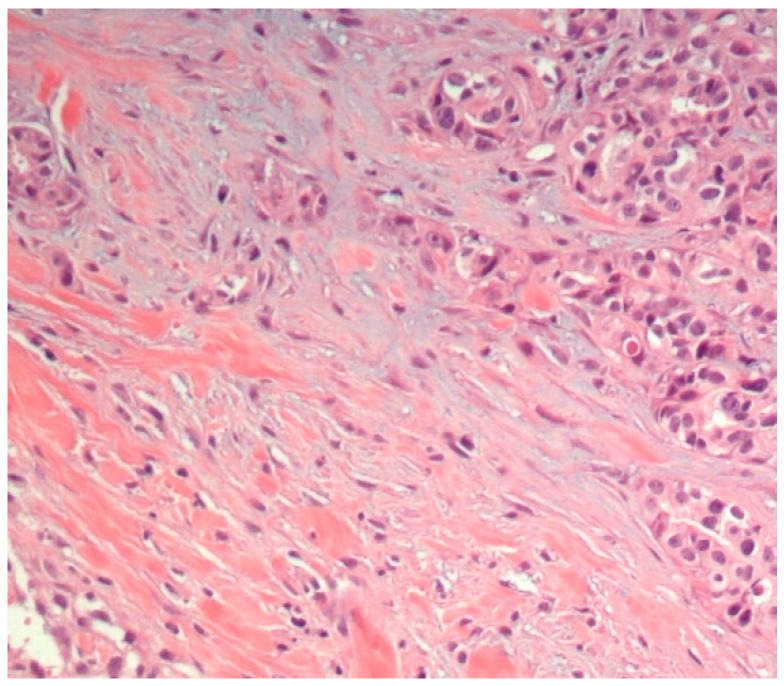
Mammary gland, complex adenoma (HE staining, 200×). There are epithelial (tubular) and myoepithelial cell propagation. The myoepithelial cells are fusiform to stellate and are surrounded by a basophilic mucinous matrix.

**Figure 2:**
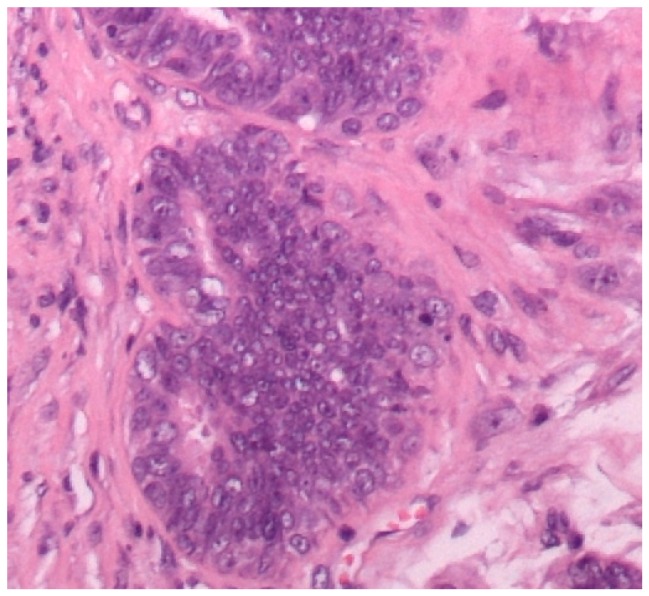
Canine mammary gland, ductal carcinoma (HE staining, 400×). The ducts are lined by a multilayered epithelium exhibiting considerable nuclear and cellular pleomorphism.

**Figure 3:**
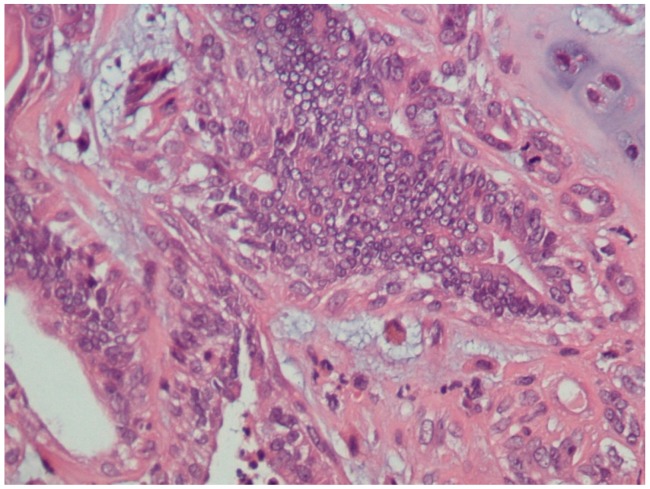
Canine mammary gland, malignant mixed mammary tumor (carcinosarcoma) (HE staining, 400×). Two neoplastic populations are present. There are neoplastic cells showing chondroid differentiation (chondrosarcoma). There is considerable nuclear and cellular pleomorphism of the epithelial cells.

A total of 23 mammary gland tumors were observed. According to Goldschmidt et al. [13], these tumors were classified as benign (4/23, 17.4%) or malignant (19/23, 82.6%). In addition, the benign tumors were subclassified according their predominant types of cells, i.e., adenoma (1/23, 4.3%), fibroadenoma (1/23, 4.3%), and complex adenoma (2/23, 8.7%) (Figure 1). Moreover, the malignant tumors were subclassified into malignant epithelial neoplasms (15/23, 65.2%) (Figure 2), malignant epithelial neoplasms—special types (Lipid-rich carcinoma) (1/23, 4.3%), malignant mesenchymal neoplasms—sarcomas (Osteosarcoma) (1/23, 4.3%), and malignant mixed mammary tumors—carcinosarcomas (2/23, 8.7%) (Figure 3).

### 3.2. Microsatellite Instability (MSI) Screening

Changes in MS markers in the CMT samples compared with the DNA of normal mammary tissue samples were recognized as alterations in the electrophoretic migration or loss of major band(s). Among 77 MS markers, MSI existed in 11, whereas 66 markers showed stability. In all the tumor-affected patients, MSI was identified at one or more loci. The highest incidence of MSI was observed in the tumor from dog no. 19, which exhibited MSI at seven loci (7/11, 63.6%), whereas only one MSI locus was observed in each of dogs no. 1, 4, 6, 7, 8, 11, 12, 16 and 22. According to this criterion, canine mammary gland tumors from nine dogs (dog no. 2, 5, 13, 14, 15, 17, 19, 21 and 23) presented MSI-H, and the rest of the canine mammary gland tumors demonstrated MSI-L in this study (Table 4).

In addition, MSI frequency for each microsatellite marker was presented in (Table 5). Among the markers, the ABC9TETRA marker showed the highest instability frequency (10/23, 43.4%) in the tumor samples. In addition, MEPIA exhibited MSI in eight tumors (8/23, 34.8%), and 9A5, SCNA11 and FJL25 exhibited MSI in seven tumors (7/23, 30.4%).

### 3.3. Mismatch Repair-Related Gene Expression

**Figure 4:**
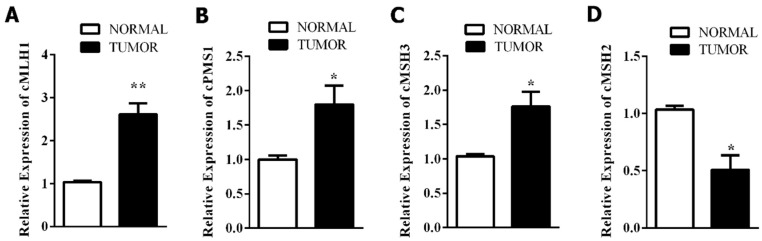
Expression of genes *cMLH1* (**A**), *cPMS1* (**B**), *cMSH3* (**C**), *cMSH2* (**D**) in normal and tumor canine mammary tissues (* *p* < 0.05, ** *p* < 0.01).

The RT-qPCR results revealed that mRNA expression of the *MMR* system genes significantly increased in CMT (Figure 4). Compared to those in the normal tissues, the genes *cMLH1* (*p* < 0.0043), *cPMS1 (p* < 0.046) and c*MSH3* (*p* < 0.026) in the tumor tissues were significantly upregulated, but c*MSH2 (p* < 0.016) was downregulated.

### 3.4. The Extent of Polymorphism in cMSH2 Gene

The sequence of *cMSH2* in the dog genome retrieved from the gene bank database of NCBI revealed a 3152 bp gene sequence consisting of 16 exons. Hence, exon-specific primers were designed and amplified from the CMT and normal DNA templates using PCR. The resulting amplicons were separated on 10% nondenaturing polyacrylamide gels. These PCR amplicons were then sequenced to determine the presence of polymorphism among the samples.

**Figure 5:**
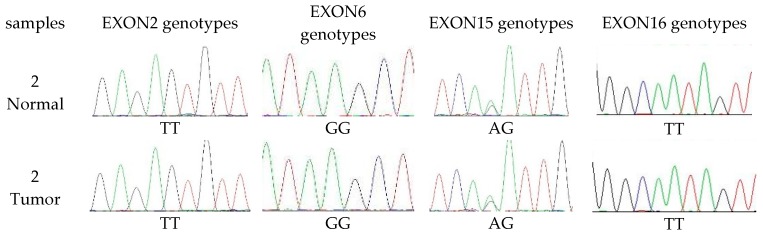

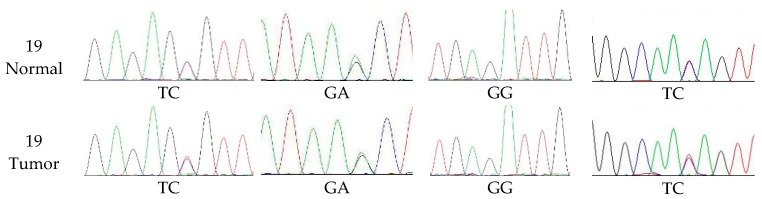
Genotypes of *cMSH2* exons in tumor and normal canine mammary samples.

The PCR amplicons obtained from the DNA templates of the tumor and normal tissues of the 23 female dogs used in this study were sequenced to identify the nature of the changes or polymorphisms. The amplified products of the 16 exons were sequenced (Figure 5). Single nucleotide polymorphisms were observed in exons number 2, 6, 15 and 16 of *cMSH2* in the sequences of all the normal and the tumor dog samples. The genotypes of exon 2 were TT (5/23, 21.7%), TC (17/23, 73.9%) and CC (1/23, 4.3%). The genotypes of exon 6 were GG (9/23, 39.1%), GA (11/23, 47.8%) and AA (3/23, 13.0%). The genotypes of exon 15 were GG (16/23, 69.6%), GA (5/23, 21.7%) and AA (2/23, 8.7%). The genotypes of exon 16 were TT (8/23, 34.8%), TC (15/23, 65.2%).

## 4. Discussion

Dogs, primarily unspayed female dogs, are frequently victims of mammary neoplasia. Lack of effective diagnostic tools during the earlier stages of carcinogenesis is a serious concern, as this disease is unresponsive to treatments at later stages [18]. To enhance the effectiveness of CMT treatment, it is essential to diagnose it in its early stages. Hence, this study hypothesized that the successful diagnosis could be made possible by employing the biomarkers that could distinguish the tumorous glands from the healthy ones. The dog marker genome map has been comprehensively developed during recent years. To date, more than 2000 microsatellite markers have been reported, and many of them have been assigned to linkage groups and specific chromosomes [4]. Some studies have reported a few biomarkers for the diagnosis and prognosis of CMT [19,20]. However, there is a lack of rapid, sensitive and specific biomarkers, which are required for the diagnosis of carcinogenesis in earlier stages.

In this study, a panel of 77 microsatellite markers was used to check for MSI, among which 11 markers showed instability and 66 markers showed uniform amplification. These results were in accordance with an earlier published study that evaluated 35 canine mammary tumors, among which 13 (37%) had stable genotypes, and 22 (63%) exhibited aberrations in 1 or 2 MS and 4 tumors (11%) demonstrated high instability, with aberrations in 29% to 61% of MS [15]. MSI was detected at one or more loci in all the tumor-affected dogs in this study. In particular, the highest frequency of MSI was observed for the tumor from dog no. 19, which had seven loci (7/11, 63.6%) affected displaying MSI-H.

Moreover, among the markers, ABC9TETRA showed the highest frequency of instability (10/23, 43.4%). In addition, MSI was also evident in MEPIA in eight tumors (8/23, 26%). Furthermore, FLJ32685, SCNA11, and 9A5 exhibited MSI in seven tumors (7/23, 30.4%). The results of this study were comparable to those of an earlier published report related to human HPNCC disease, which used 21 patients and analyzed 78 tumor samples for MSI by microsatellite markers. They classified 26.9% tumors as high MSI (MSI-H), 11 (14.1%) as low instability (MSI-L) and 46 patients (59%) as medium instability (MS-S) [21]. Similarly, the findings of the present study are in agreement with those of Ando et al. [22], who showed a higher frequency of MSI-L in human breast tumors. The results of this study were also in accordance with another study, which demonstrated MSI-L in grade III ductal and lobular breast cancers [23]. Furthermore, Yee et al. [24] also observed a high frequency of MSI-H in human breast cancers.

The RT-qPCR-based findings showed upregulation of some *MMR* genes (c*MSH3*, c*MLH1*, c*PMSI*), but downregulation of c*MSH2*, compared to the levels in the normal group. The expression of c*MSH2* has been associated with tumor development in studies on gastric cancers [25], glioblastomas [26], salivary gland tumors [27], malignant melanomas [28], ovarian carcinomas [29], urothelial carcinomas [30], and endometrial carcinomas [31]. In addition, higher expression of *MMR* genes could be acquired by a genetic change, followed by an alteration in gene expression, thereby leading to an increased level of *MMR* proteins with impaired functions [32]. Since tumor cells acquire comprehensive genetic changes, increased levels of *MMR* gene expression could hence be associated with a cellular adaptation aimed at repairing the DNA lesions [33]. Moreover, the overexpression of *MMR* genes in the cancerous cells could represent the response to the fast-growing number of replication errors in a tissue with an increased rate of cell divisions [34]. Furthermore, germline mutations in DNA *MMR* genes, particularly in *MLH1* and c*MSH2* genes, were associated with tumor development in humans in earlier studies [35,36]. Due to the observation of lower mRNA expression of the c*MSH2* gene, the present study further investigated its role in the genesis of CMT. The results of the present study confirmed polymorphisms in the c*MSH2* gene in exons 2, 6, 15 and 16.

In conclusion, all CMT demonstrated MSI using the eleven microsatellite markers selected in this study, with eight tumors presenting MSI-H *MMR* gene abnormalities, such as overexpression of *cMSH3*, *cMLH1*, and *cPMSI* and SNPs in the *cMSH2* gene, were also found in these tumors. MSI may be related to MMR genes abnormalities and may be used as diagnostic tools for the CMT in the future.

## Figures and Tables

**Table 1:** List of canine DNA primer pairs used for PCR-amplification along with approximate product size and annealing temperature [14].

CODE	Primer Forward 5′→3′	Primer Reversed 5′→3′	Expected Size bp
FLJ32685	CTGCCTCAGCTGGGAAAATA	CACTACAGCTGGGATCAGCA	433
SCNA11	GCAGTTTGGGGCTGCTAAA	AGAATGGAATCTTGCCCAGA	267
ABC9TETRA	GCATTAAGGAGGGCACTTGA	GACCCAGCCTTGAAAGAATG	220
SCNA10	TCCAAGCATCCTCTTATCCA	CCACGTTGGTCTCCCTACTTA	196
ANGPT1	GTTTTCCTGCTGTCCCAGTG	TTCCCTTTTGTGAATCCTGC	414
FLJ20511	AAAGGCAGTCAACCAGTCC	CTGTGCAGTTTGCGGAGTAC	403
IGHE	CAAGACTGGCTCTGCTCTG	CCACTGAAAACAAGCCCATC	140
PPP1R9A	TAAAGATCCAAGTGGCGAGG	AACCACTCCCTTCACCACAG	189
MEPIA	GGTTCTGGGATCAAGTTCCA	CTGGTGGTTTCCTCTCCCTA	345
CDH4	AAGTCAACAAGCTCCATCCC	AGGATTTTCCCCTAAGAGCTG	142
9A5	CATGCAGATGCCCCTAATCT	GGTGACAGGTGATTCTTGGA	173

**Table 2:** Primer used for real time PCR.

No.	Name of GENE	Primer Sequence	Tm (C)	Amplicon Size
1.	cMSH2	F: CATTGGTGTCGTGGGTGTTA R: CAAAGTCCTAGCTTCCTCTGTATG	62	96
2.	cMSH3	F: CCTCGTGGCAAAGGGATATAA R: TTTCCGGGAGAACAGTGAAC	62	100
3.	cMLH1	F: GAGGGTCTGCCTATCTTCATTC R: GCACATTCTTTACTGAGGCTTTC	62	92
4.	cPMS1	F: CAGCAGTCGAGTAGTCAAGAAA R: GCATCCTCCAAACTGGTCTTA	62	105
5.	GAPDH	F: GATGCTGGTGCTGAGTATGT R: CAGAAGGAGCAGAGATGATGAC	62	112

**Table 3:** Primers used to amplify exon of *cMSH2* gene.

Primer	Sequence (5′–3′)	Size of Fragments Amplified (bp)
MSH2 exon1 F MSH2 exon1 R	GGACGCTCCGAAATGG GTCCACTCCCGCCCCT	235
MSH2 exon2 F MSH2 exon2 R	TGAGAGAAGAATGTAGGTTGGGG GCACACAATAGAATTCCCTCACA	333
MSH2 exon3 F MSH2 exon3 R	ATTGTGTATAAATCCAGCTGCCA CTTCATCCCTACCTTGATTCCCT	421
MSH2 exon4 F MSH2 exon4 R	TGGATTGGTTTGTTATGCTGTTGT TCACAAGCTTCGTCACAGTAAGA	385
MSH2 exon5 F MSH2 exon5 R	TGAAACAAGGTACCAGCATCTC TTACGCTTCTTAATTGTATTCTTCA	443
MSH2 exon6 F MSH2 exon6 R	TGGCACAGTAAGGTTTTCACTAA GATCAAGTGGCATAATCCTAGAGT	269
MSH2 exon7 F MSH2 exon7 R	TAATCCCAGTGCAATTTATTTCAGA CCCAACTTTATAAGGACAGCACA	299
MSH2 exon8 F MSH2 exon8 R	GAGACTTGCTGCGCTATTTGT TTCAAAAATACTTTGCTGCTGAAT	276
MSH2 exon9 F MSH2 exon9 R	ATTGTTATTTCCATCTTTACCCATC GAATTACTCAAACCACCAATGAG	216
MSH2exon10F MSH2exon10R	CTGTAGACATCTATGACCTTTTTCT GGAACATGCACATTTCATCCGAG	277
MSH2exon11F MSH2exon11R	GCTTATAGGACAGATGCTCTGGG TGCCTTGTAGCTCTTGGGTG	832
MSH2exon12F MSH2exon12R	TCAGTATTCCTGTGCACATTTTCT AAGCCCATAATTTAGGTGGGG	323
MSH2exon13F MSH2exon13R	TTTGGCAGTTAATGGTTCTGCTT CAGTCTGAGGGGACTGGGAAAT	374
MSH2exon14F MSH2exon14R	TGTCCCTTAACACATCTTTCCC CCAGTCACGCCCGAATTTAC	399
MSH2exon15F MSH2exon15R MSH2exon16F MSH2exon16R	GACAAGGTGAGGTGAACACG TCACACAGGAACAAATAACTCATC TGGTCAACTTAGGACTTTCTGTAA CCTTGGCTGCGACTTGTTTTT	346 629

**Table 4:** Histological classification and MSI markers in canine mammary gland tumors.

Dog No.	Age of Dog	Breed	Tumor Histo-Type	Marker	Number of MSI
1	6	Shish Tzu	Complex adenoma	CDH4	1/11
2	7	Pomeranian	Malignant epithelial neoplasms-Tubulopapillary carcinoma	ABC9TETRA, ANGPT1, PPP, MEPIA	4/11
3	8	Shish Tzu	Malignant epithelial neoplasms-Solid carcinoma	9A5, SCNA10	2/11
4	8	Hybrid	Fibroadenoma	ANGPT1	1/11
5	9	Poodle	Malignant epithelial neoplasms-Complex carcinoma	9A5, ABC9TETRA, FLJ20511, IGHE, FLJ32685	5/11
6	9	Pomeranian	Adenoma	FLJ20511	1/11
7	9	Hybrid	Malignant epithelial neoplasms-special types (Lipid-rich carcinoma)	SCNA10	1/11
8	10	Shish Tzu	Complex adenoma	ABC9TETRA	1/11
9	10	Poodle	Malignant Mesenchymal neoplasms-osteosarcomas	ABC9TETRA, FLJ20511, SCNA10	3/11
10	10	Poodle	Malignant epithelial neoplasms-Tubulopapillary carcinoma	MEPIA, IGHE	2/11
11	11	Poodle	Malignant epithelial neoplasms-Ductal carcinoma	PPP	1/11
12	11	Poodle	Malignant epithelial neoplasms-Ductal carcinoma	FLJ32685	1/11
13	11	Pomeranian	Malignant epithelial neoplasms-Ductal carcinoma	ABC9TETRA, ANGPT1, SCNA10, SCNA11, MEPIA	5/11
14	11	Poodle	malignant mixed mammary tumor—carcinosarcoma	9A5, FLJ32685, SCNA11, MEPIA	4/11
15	11	Pomeranian	Malignant epithelial neoplasms-Complex carcinoma	ABC9TETRA, FLJ20511, FLJ32685, SCNA11	4/11
16	12	Poodle	Malignant epithelial neoplasms-Tubular carcinoma	SCNA11	1/11
17	12	Poodle	Malignant epithelial neoplasms-Ductal carcinoma	ANGPT1, IGHE, SCNA10, SCNA11, MEPIA, CDH4	6/11
18	12	Hybrid	Malignant epithelial neoplasms-Tubular carcinoma	9A5, CDH4, PPP	3/11
19	13	Poodle	Malignant epithelial neoplasms-Ductal carcinoma	9A5, ABC9TETRA, FLJ20511, FLJ32685, SCNA11, PPP, MEPIA	7/11
20	13	Poodle	malignant mixed mammary tumor—carcinosarcoma	ABC9TETRA, 9A5	2/11
21	15	Hybrid	Malignant epithelial neoplasms-Tubulopapillary carcinoma	9A5, FLJ20511, SCNA10, CDH4, MEPIA,	5/11
22	15	Poodle	Malignant epithelial neoplasms-Ductal carcinoma	ABC9TETRA	1/11
23	15	Poodle	Malignant epithelial neoplasms-Ductal carcinoma	ABC9TETRA, FLJ20511, SCNA11, PPP, MEPIA	5/11

**Table 5:** Frequency of MSI for each microsatellite marker.

Markers	Tumor Cases	Frequency	Rate of Change
ABC9TETRA	2, 5, 8, 9, 13, 15, 19, 20, 22, 23	10/23	43.4%
MEPIA	2, 10, 13, 14, 17, 19, 21, 23	8/23	34.8%
9A5	3, 5, 14, 18, 19, 20, 21	7/23	30.4%
SCNA 11	13, 14, 15, 16, 17, 19, 23	7/23	26.1%
FLJ20511	5, 6, 9, 15, 19, 21, 23	7/23	26.1%
SCNA10	3, 7, 9, 13, 17, 21	6/23	26.1%
FLJ32685	5, 12, 14, 15, 19	5/23	21.7%
PPP	2, 11, 18, 19, 23	5/23	21.7%
ANGPT1	2, 4, 13, 17	4/23	17.4%
CDH4	1, 17, 18, 21	4/23	17.4%
IGHE	5, 10, 17	3/23	13.0%

## Abbreviations

HE	Hematoxylin and eosin
MMR	Mismatch repair
CMT	Gland tumor
CI	Chromosomal instability

## References

[1] Shen Z. (2011). Genomic instability and cancer: An introduction. J. Mol. Cell Biol..

[2] Thibodeau S.N., Bren G., Schaid D. (1993). Microsatellite instability in cancer of the proximal colon. Science.

[3] Parsons R., Li G.M., Longley M.J., Fang W.H., Papadopoulos N., Jen J., de la Chapelle A., Kinzler K.W., Vogelstein B., Modrich P. (1993). Hypermutability and mismatch repair deficiency in RER+ tumor cells. Cell.

[4] Klukowska J., Strabel T., Mackowski M., Switonski M. (2003). Microsatellite polymorphism and genetic distances between the dog, red fox and arctic fox. J. Anim. Breed. Genet..

[5] Kunkel T.A., Erie D.A. (2005). DNA mismatch repair. Annu. Rev. Biochem..

[6] Schofield M.J., Hsieh P. (2003). DNA mismatch repair: Molecular mechanisms and biological function. Annu. Rev. Microbiol..

[7] Conde J., Silva S.N., Azevedo A.P., Teixeira V., Pina J.E., Rueff J., Gaspar J.F. (2009). Association of common variants in mismatch repair genes and breast cancer susceptibility: A multigene study. Bmc Cancer.

[8] Niessen R.C., Berends M.J., Wu Y., Sijmons R.H., Hollema H., Ligtenberg M.J., de Walle H.E., de Vries E.G., Karrenbeld A., Buys C.H. (2006). Identification of mismatch repair gene mutations in young patients with colorectal cancer and in patients with multiple tumours associated with hereditary non-polyposis colorectal cancer. Gut.

[9] Kane M.F., Loda M., Gaida G.M., Lipman J., Mishra R., Goldman H., Jessup J.M., Kolodner R. (1997). Methylation of the hMLH1 promoter correlates with lack of expression of hMLH1 in sporadic colon tumors and mismatch repair-defective human tumor cell lines. Cancer Res..

[10] Koopman M., Kortman G.A.M., Mekenkamp L., Ligtenberg M.J.L., Hoogerbrugge N., Antonini N.F., Punt C.J.A., Van K.J.H.J.M. (2009). Deficient mismatch repair system in patients with sporadic advanced colorectal cancer. Br. J. Cancer.

[11] Poplawski T., Zadrozny M., Kolacinska A., Rykala J., Morawiec Z., Blasiak J. (2005). Polymorphisms of the DNA mismatch repair gene HMSH2 in breast cancer occurence and progression. Breast Cancer Res. Treat..

[12] Mik M., Dziki L., Malinowska K., Trzcinski R., Majsterek I., Dziki A. (2017). Polymorphism of MSH2 Gly322Asp and MLH1 -93G>A in non-familial colon cancer—A case-controlled study. Arch. Med. Sci. AMS.

[13] Goldschmidt M., Pena L., Rasotto R., Zappulli V. (2011). Classification and grading of canine mammary tumors. Vet. Pathol..

[14] Litt M., Bestwick M., Winther M., Jakobs P. (2005). Fifty-four new gene-based canine microsatellite markers. J. Hered..

[15] McNiel E.A., Griffin K.L., Mellett A.M., Madrill N.J., Mickelson J.R. (2007). Microsatellite instability in canine mammary gland tumors. J. Vet. Intern. Med..

[16] Jouquand S., Priat C., Hitte C., Lachaume P., Andre C., Galibert F. (2000). Identification and characterization of a set of 100 tri- and dinucleotide microsatellites in the canine genome. Anim Genet..

[17] Imai K., Yamamoto H. (2008). Carcinogenesis and microsatellite instability: The interrelationship between genetics and epigenetics. Carcinogenesis.

[18] Merlo D.F., Rossi L., Pellegrino C., Ceppi M., Cardellino U., Capurro C., Ratto A., Sambucco P.L., Sestito V., Tanara G. (2008). Cancer incidence in pet dogs: Findings of the Animal Tumor Registry of Genoa, Italy. J. Vet. Intern. Med..

[19] Sunil Kumar B.V., Bhardwaj R., Mahajan K., Kashyap N., Kumar A., Verma R. (2018). The overexpression of Hsp90B1 is associated with tumorigenesis of canine mammary glands. Mol. Cell. Biochem..

[20] Pandey M., Kumar B.V., Singh S., Verma R. (2017). Development of recombinant matrix metalloproteinase-3 based sandwich ELISA for sero-diagnosis of canine mammary carcinomas. J. Immunoass. Immunochem..

[21] Loukola A., Eklin K., Laiho P., Salovaara R., Kristo P., Järvinen H., Mecklin J.-P., Launonen V., Aaltonen L.A. (2001). Microsatellite marker analysis in screening for hereditary nonpolyposis colorectal cancer (HNPCC). Cancer Res..

[22] Ando Y., Iwase H., Ichihara S., Toyoshima S., Nakamura T., Yamashita H., Toyama T., Omoto Y., Karamatsu S., Mitsuyama S. (2000). Loss of heterozygosity and microsatellite instability in ductal carcinoma in situ of the breast. Cancer Lett..

[23] Halford S.E., Sawyer E.J., Lambros M.B., Gorman P., Macdonald N.D., Talbot I.C., Foulkes W.D., Gillett C.E., Barnes D.M., Akslen L.A. (2003). MSI-low, a real phenomenon which varies in frequency among cancer types. J. Pathol..

[24] Yee C.J., Roodi N., Verrier C.S., Parl F.F. (1994). Microsatellite instability and loss of heterozygosity in breast cancer. Cancer Res..

[25] Li M., Liu L., Wang Z., Wang L., Liu Z., Xu G., Lu S. (2008). Overexpression of hMSH2 and hMLH1 protein in certain gastric cancers and their surrounding mucosae. Oncol. Rep..

[26] Srivastava T., Chattopadhyay P., Mahapatra A.K., Sarkar C., Sinha S. (2004). Increased hMSH2 protein expression in glioblastoma multiforme. J. Neuro-Oncol..

[27] Castrilli G., Fabiano A., La Torre G., Marigo L., Piantelli C., Perfetti G., Ranelletti F.O., Piantelli M. (2002). Expression of hMSH2 and hMLH1 proteins of the human DNA mismatch repair system in salivary gland tumors. J. Oral Pathol. Med..

[28] Hussein M.R., Sun M., Roggero E., Sudilovsky E.C., Tuthill R.J., Wood G.S., Sudilovsky O. (2002). Loss of heterozygosity, microsatellite instability, and mismatch repair protein alterations in the radial growth phase of cutaneous malignant melanomas. Mol. Carcinog..

[29] Friedrich M., Villena-Heinsen C., Meyberg R., Woll-Hermann A., Reitnauer K., Schmidt W., Tilgen W., Reichrath J. (1999). Immunohistochemical analysis of DNA ‘mismatch-repair’ enzyme human Mut-S-Homologon-2 in ovarian carcinomas. Histochem. J..

[30] Leach F.S., Hsieh J.T., Molberg K., Saboorian M.H., McConnell J.D., Sagalowsky A.I. (2000). Expression of the human mismatch repair gene hMSH2: A potential marker for urothelial malignancy. Cancer.

[31] Hamid A.A., Mandai M., Konishi I., Nanbu K., Tsuruta Y., Kusakari T., Kariya M., Kita M., Fujii S. (2002). Cyclical change of hMSH2 protein expression in normal endometrium during the menstrual cycle and its overexpression in endometrial hyperplasia and sporadic endometrial carcinoma. Cancer.

[32] Berger M.F., Lawrence M.S., Demichelis F., Drier Y., Cibulskis K., Sivachenko A.Y., Sboner A., Esgueva R., Pflueger D., Sougnez C. (2011). The genomic complexity of primary human prostate cancer. Nature.

[33] Lapointe J., Li C., Giacomini C.P., Salari K., Huang S., Wang P., Ferrari M., Hernandez-Boussard T., Brooks J.D., Pollack J.R. (2007). Genomic profiling reveals alternative genetic pathways of prostate tumorigenesis. Cancer Res..

[34] Burger M., Denzinger S., Hammerschmied C.G., Tannapfel A., Obermann E.C., Wieland W.F., Hartmann A., Stoehr R. (2006). Elevated microsatellite alterations at selected tetranucleotides (EMAST) and mismatch repair gene expression in prostate cancer. J. Mol. Med. (Berl. Ger. ).

[35] Zahary M.N., Kaur G., Abu Hassan M.R., Singh H., Naik V.R., Ankathil R. (2012). Germline mutation analysis of MLH1 and MSH2 in Malaysian Lynch syndrome patients. World J. Gastroenterol. WJG.

[36] Miyaki M., Konishi M., Tanaka K., Kikuchi-Yanoshita R., Muraoka M., Yasuno M., Igari T., Koike M., Chiba M., Mori T. (1997). Germline mutation of MSH6 as the cause of hereditary nonpolyposis colorectal cancer. Nat. Genet..

